# *LRP1* and *RAGE* Expression in the Frontal Cortex in the Alzheimer’s Disease Ischemia Model During 2 Years of Follow-Up

**DOI:** 10.3390/ijms27135831

**Published:** 2026-06-28

**Authors:** Ryszard Pluta, Marzena Ułamek-Kozioł, Janusz Kocki, Anna Bogucka-Kocka, Stanisław J. Czuczwar, Jacek Bogucki

**Affiliations:** 1 Department of Pathophysiology, Medical University of Lublin, 20-090 Lublin, Poland; czuczwarsj@yahoo.com; 2 Department of Neurology, Institute of Psychiatry and Neurology, 02-957 Warsaw, Poland; marzena_ulamek@wp.pl; 3 Department of Clinical Genetics, Medical University of Lublin, 20-080 Lublin, Poland; janusz.kocki@tlen.pl; 4 Department of Biology and Genetics, Medical University of Lublin, 20-093 Lublin, Poland; anna.kocka@tlen.pl; 5 Faculty of Medicine, John Paul II Catholic University of Lublin, 20-708 Lublin, Poland; jacekbogucki@wp.pl

**Keywords:** brain ischemia, Alzheimer’s disease, frontal cortex, *LRP1*, *RAGE*, genes, amyloid, tau protein, transport, model

## Abstract

Exploration of the gene-level changes that occur during post-ischemic neurodegeneration in the frontal cortex is crucial for understanding the development of dementia. An ischemic model of Alzheimer’s disease was used to evaluate changes in the expression of the receptor for advanced glycation end products (*RAGE*) and low-density lipoprotein receptor-related protein 1 (*LRP1*), which are associated with amyloid and tau protein, in the frontal cortex after 10 min of cerebral ischemia, with survival at 2, 7, and 30 days and 0.5, 1, 1.5, and 2 years. *LRP1* and *RAGE* expression was assessed by reverse transcription-quantitative polymerase chain reaction. After two days and 1.5 and 2 years post-ischemia, *LRP1* expression was increased, after 7 days and 0.5 years it was decreased, and after 30 days and 1 year it oscillated around control values. The decrease in *RAGE* expression was statistically significant compared to the control group after 2 and 7 days and after 0.5 years, and after 30 days it oscillated around the control value, while after 1–2 years it increased significantly. *RAGE* and *LRP1* expression showed the same pattern of changes from day 7 to year 2, peaking at 1 and 1.5 years, respectively. Another peak of *RAGE* overexpression was noted 2 years after ischemia. After 1, 1.5 and 2 years, overexpression of *RAGE* and *LRP1* was observed after ischemia, with the dynamics of *LRP1* changes being lower. Overall, the data showed a predominance of *RAGE* expression over *LRP1* expression at 1-, 1.5-, and 2-years post-ischemia. The modification of *LRP1* and *RAGE* after ischemia is useful in studying the molecular ischemic pathways involved in the development of Alzheimer’s disease.

## 1. Introduction

Despite years of research into the etiology of Alzheimer’s disease, based on the role of amyloid and tau protein as disease triggers, these studies have been hampered because they have failed to resolve the problem and, most importantly, have not led to definitive conclusions. Due to the ineffectiveness of these studies and pressure from patients and their families affected by Alzheimer’s disease, the scientific community has undergone a shift in its approach to understanding the causes of this condition. This situation inspired a group of scientists who have been studying cerebral ischemia for years to focus on the changes occurring post-ischemia, which—similar to Alzheimer’s disease—dominate in the hippocampus, leading to the development of amyloid plaques, neurofibrillary tangles, and dementia [1,2,3,4,5,6,7,8,9,10,11,12,13]. In this context, it has been proposed that cerebral ischemia may play a key role in fueling amyloid and tau protein pathology in the development of Alzheimer’s disease. This approach has been supported and validated by numerous experimental and clinical studies [4,7,8]. Recent advances in research on the ischemic etiology of Alzheimer’s disease have revealed dysregulation of genes associated with Alzheimer’s disease, including secretases, amyloid precursor protein, apoptosis, autophagy, mitophagy, tau protein, α-synuclein, apolipoproteins, LRP1, and RAGE [4,7]. A link has been demonstrated between genes whose dysregulation is a consequence of cerebral ischemia and the cellular and tissue neuropathology and their proteins typical of Alzheimer’s disease [7]. These observations have clearly demonstrated that, following cerebral ischemia, changes occur in the expression of Alzheimer’s disease-associated genes and in the folding proteins such as amyloid, tau protein, and α-synuclein [8,9]. This has been shown to lead to massive neuronal death, disruption of the neuronal network and brain atrophy, finally leading to the development of Alzheimer’s disease-type dementia. Current data suggest common genomic and proteomic factors in cerebral ischemia and Alzheimer’s disease, as well as a long-term conversion of brain ischemia neurodegeneration to Alzheimer’s disease [7,8]. It appears that the ischemia model of Alzheimer’s disease may be useful in defining the role of folding proteins and genes dysregulation in Alzheimer’s disease.

In recent years, brain ischemia in rats and mice has become one of the important models for studying the neuropathogenesis of Alzheimer’s disease [1,2,3,4,5,6,7]. These models mimic Alzheimer’s disease, showing amyloid accumulation in the form of diffuse and senile plaques [8,9], tau protein hyperphosphorylation [10,11,12,13], neuroinflammation [14,15], cerebral amyloid angiopathy [14,16], neuronal death and brain atrophy [9,17], decreased acetylcholine levels [1,3], and impairments in learning and memory with the development of full-blown dementia [5,6,18,19,20,21]. These observations indicate that ischemia drives the formation and accumulation of amyloid and tau protein, which are characteristic of Alzheimer’s disease. Studies also indicate the importance of ischemic blood–brain barrier dysfunction in the progression of post-ischemic neurodegeneration, such as in Alzheimer’s disease [16,22,23]. Thus, tau protein, amyloid, and recurrent hypoperfusion constitute mechanistic links between typical features of Alzheimer’s disease and the post-ischemic brain [2,3,4,5,7,22].

Alzheimer’s disease is characterized by the selective susceptibility of a specific population of neurons to pathological factors, starting from the hippocampus, similar to cerebral ischemia [9,24,25,26]. Additionally, it has been suggested that factors such as the presence of certain positive divalent cations [27] or ionic strength [28] may induce amyloid formation and the progression of Alzheimer’s disease. It is known that the neuropathological progression of Alzheimer’s disease does not proceed uniformly throughout the brain but shows significant regional specificity [24,26]. The neuropathological changes in Alzheimer’s disease are believed to begin in the hippocampus and entorhinal cortex and then gradually spread to the frontal, parietal, and temporal cortex of the brain [29]. Despite many years of research, the genes and molecular mechanisms underlying the neuropathological development of Alzheimer’s disease in different brain structures are still not well understood [25,26,30,31,32].

Amyloid and tau protein are characteristic of Alzheimer’s disease. It is important to note that amyloid and tau protein in the brain are regulated by transmembrane proteins, namely the receptor for advanced glycation end products (RAGE) and low-density lipoprotein receptor-related protein 1 (LRP1). LRP1 has been shown to exert neuroprotective effects in the neuropathology of Alzheimer’s disease by improving amyloid clearance across the blood–brain barrier and increasing tau protein proteolysis [23,33,34,35,36]. LRP1 reduces amyloid production by competing with amyloid precursor protein for metabolism by β- and γ-secretase in neuronal cell lines [35,37]. Moreover, LRP1 is a key regulator of tau protein metabolism through increased internalization, which facilitates its degradation by lysosomes [35,38]. LRP1 exerts neuroprotective effects in Alzheimer’s disease by activating platelet-derived growth factor signaling [35]. LRP1 is a key regulator of amyloid homeostasis and accumulation and tau protein uptake and spreading in Alzheimer’s disease [34,39]. Nevertheless, accumulating evidence indicates that LRP1 not only regulates the neuropathogenesis of Alzheimer’s disease but also maintains brain homeostasis in an amyloid-independent manner [34].

On the other hand, RAGE has the opposite effect [36]. RAGE plays a key role in Alzheimer’s disease by influencing amyloid production and accumulation, neurofibrillary tangle formation, impaired synaptic transmission, and neuronal degeneration [35,40]. RAGE is a significant contributor to amyloid production by increasing β- and γ-secretase activity and activating the neuroinflammatory response and oxidative stress [35,40]. In addition, RAGE acts as an important transporter, regulating the influx of amyloid from the circulatory system into the brain. RAGE causes dysfunction of neuronal circuits that constitute both the functional and structural basis of cognitive impairment. In addition, RAGE initiates amyloid-dependent tau protein hyperphosphorylation, which is also associated with cognitive impairment [35,40]. RAGE’s interaction with amyloid impairs the brain’s ability to clear it, leading to increased amyloid accumulation and increased neuronal damage [41]. This additionally increases neuroinflammatory responses and oxidative stress, ultimately leading to progressive neurodegeneration with age [41].

At different stages of Alzheimer’s disease, different brain structures are affected differently [29]. Selective vulnerability of specific brain structures is a fundamental feature of neurodegenerative diseases, including Alzheimer’s disease and brain ischemia. However, the common genomic and proteomic processes in Alzheimer’s disease and post-ischemic brain injury, which originate in the hippocampus and spread to other brain regions, are still not well understood. Various methods have been used to predict the course of Alzheimer’s disease, but they have never focused on examining different brain structures at different times. Understanding the changes occurring in different brain structures is essential to explaining the neuropathological mechanisms in the early and late stages of Alzheimer’s disease. The overall goal of our research is to investigate gene changes associated with Alzheimer’s disease in various brain regions following ischemia. In this article, we continue our research limited to long-term gene changes in the frontal cortex in an ischemic model of Alzheimer’s disease.

It has been previously shown that an ischemic episode in the frontal cortex causes a series of harmful phenomena that can last from a few minutes to a lifetime [9,17,42,43,44,45]. It has been revealed that neuronal death in the frontal cortex after ischemia is associated not only with excitotoxicity but also with the neurotoxicity of amyloid and tau protein [43,44,45]. Alzheimer’s disease-associated amyloid and tau protein and their genes have been shown to play a significant role in progressive and irreversible neurodegeneration in the ischemic frontal cortex [43,44,45]. Furthermore, chronic dysfunction of the blood–brain barrier causes amyloid and tau protein to leak from the blood into the cortex [16,46]. Long-term monitoring of the ischemic frontal cortex revealed acute and chronic neuronal changes and progressive neuronal death [9,17,42]. Apoptosis, autophagy, and mitophagy genes have been shown to be associated with neurodegenerative changes in the frontal cortex post-ischemia [43,44,45]. Activated astrocytes and microglia in the frontal cortex induce neuroinflammation that progresses over 2 years of follow-up [14,15,42]. Immunohistochemical staining of the brain after ischemia, with survival up to 1 year, showed the presence of amyloid around blood vessels and in neurons [9].

For over a decade, we have been trying to precisely define the region-specific gene expression changes that occur following brain ischemia and are associated with Alzheimer’s disease. This approach potentially provides a basis for understanding the neuropathogenesis of Alzheimer’s disease and the subsequent development of targeted therapies. Our previous experimental studies on *LRP1* and *RAGE* in the ischemic CA3 region of the hippocampus showed that both genes are involved in amyloid and tau protein pathology, which was manifested in the early post-ischemic period by *RAGE* overexpression and in the late period by *LRP1* overexpression [47]. However, the exact role of *LRP1* and *RAGE* involved in amyloid and tau protein pathology in the frontal cortex after ischemia in the Alzheimer’s disease ischemia model has not been fully elucidated. Therefore, this article presents *LRP1* and *RAGE* expression in the frontal cortex in an ischemic model of Alzheimer’s disease. The aim of this work is to continue the study of the quantitative assessment of genes in the frontal cortex associated with Alzheimer’s disease using RT-PCR, i.e., *RAGE* and *LRP1* involved in amyloid and tau protein pathology in rats that survived 2, 7, and 30 days and 0.5, 1, 1.5, and 2 years after experimental complete brain ischemia.

## 2. Results

### 2.1. LRP1 Changes Post-Ischemia

**Figure 1:**
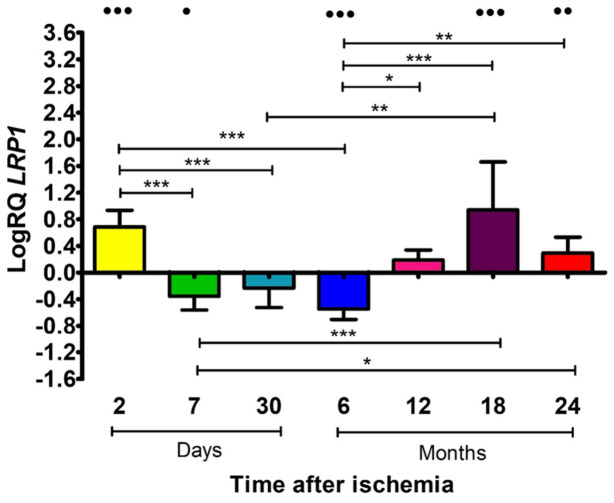
The *LRP1* changes in the frontal cortex at various recirculation times after cerebral ischemia. There were 10 samples per/time in each post-ischemia and control group. Mean values are marked; SD, standard deviation; Kruskal–Wallis test. * *p* ≤ 0.05, ** *p* ≤ 0.01, *** *p* ≤ 0.001. The dots at the top of the graph show the significance of the change on a given day between the sham group and the ischemic group using the Z test. • *p* ≤ 0.05, •• *p* ≤ 0.01, ••• *p* ≤ 0.001.

*LRP1* encodes the low-density lipoprotein receptor-related protein-1. LRP1 is considered a neuroprotective molecule in the brain following ischemic injury. After two days and 1.5 and 2 years post-ischemia, *LRP1* expression was increased, after 7 days and 0.5 years it was decreased, and after 30 days and 1 year it oscillated around control values (Figure 1). Gene expression shown in the figures is depicted using a logarithmic formula. On the 2nd day after ischemia, the median was 0.641, the minimum was 0.413, the maximum was 1.195, and the mean was 0.686. On the 7th day post-ischemia, the median was −0.420, the minimum was −0.638, the maximum was −0.042, and the mean was −0.354. After 30 days, the median was −0.092-fold, the minimum was −1.010, the maximum was −0.061, and the mean was −0.231. Half a year following ischemic injury, the median was −0.551, the minimum was −0.860, the maximum was −0.353, and the mean was −0.546. One and 1.5 years post-ischemia, the median was 0.100 and 0.943, respectively. The minimum was 0.066 and 0.127, the maximum was 0.460 and 1.817, and the mean was 0.187 and 0.940, respectively. After 2 years following ischemic injury, the median was 0.208, the minimum was 0.070, the maximum was 0.711, and the mean was 0.293. Figure 1 shows the mean data of *LRP1* expression and statistically significant differences at various recirculation times after cerebral ischemia. Figure 1 also shows statistically significant differences in *LRP1* expression values between the study and control groups at different times after brain injury caused by ischemia and reperfusion. Table 1 presents the cumulative data on the expression of *LRP1* at different times after cerebral ischemia.

### 2.2. RAGE Changes Post-Ischemia

**Figure 2:**
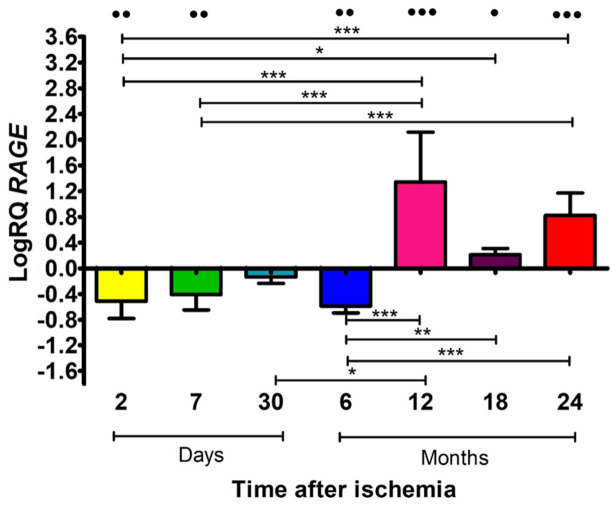
The *RAGE* changes in the frontal cortex at various recirculation times after cerebral ischemia. There were 10 samples per/time in each post-ischemia and control group. Mean values are marked; SD, standard deviation; Kruskal–Wallis test. * *p* ≤ 0.05, ** *p* ≤ 0.01, *** *p* ≤ 0.001. The dots at the top of the graph show the significance of the change on a given day between the sham group and the ischemic group using the Z test. • *p* ≤ 0.05, •• *p* ≤ 0.01, ••• *p* ≤ 0.001.

*RAGE* encodes the receptor for advanced glycation end products. RAGE is believed to have harmful influence on the brain post-ischemia. On the 2nd day after ischemia, the median expression was −0.460-fold, the minimum −0.943, the maximum −0.181 and the mean −0.511. Seven and 30 days and 0.5 years after ischemia, the median values were −0.416 (minimum −0.824 and maximum −0.080), −0.093 (minimum −0.314 and maximum −0.041), and −0.561 (minimum −0.735 and maximum −0.441), respectively. In the above post-ischemic survival times, the mean results were: −0.407, −0.135 and −0.586. The highest value of *RAGE* was noted at 1-year post-ischemia, with a median 1.749, and the minimum 0.268 and maximum 2.178 with a mean change of 1.342. One and a half to 2 years after ischemia, *RAGE* was still overexpressed, but at lower values; medians were 0.210 and 0.762, minimum and maximum were 0.062/0.377 and 0.316/1.401 and mean values were 0.212 and 0.823, respectively. Figure 2 shows the mean data of *RAGE* expression and statistically significant differences at various recirculation times after cerebral ischemia. Figure 2 also shows statistically significant differences in *RAGE* expression values between the study and control groups at different times after brain injury caused by ischemia and reperfusion. Table 1 presents the cumulative data on the expression of *RAGE* at different times after cerebral ischemia.

## 3. Discussion

In previous studies of the frontal cortex post-ischemia, we have shown changes in the expression of the following genes: amyloid precursor protein, α-secretase, β-secretase, presenilin 1 and 2, tau protein (MAPT), α-synuclein (SNCA), autophagy, mitophagy, caspase 3, apolipoprotein A1, J, and E [43,44,45]. However, this study demonstrated, for the first time, changes in *LRP1* and *RAGE* expression in the frontal cortex of an ischemic model of Alzheimer’s disease [2,4,7,8] at follow-up periods of 2, 7, and 30 days, and 0.5, 1, 1.5, and 2 years. *RAGE* expression during early post-ischemic survival (from 2 days to 6 months) was lower than control values, but at later stages (from 12 to 24 months) it exceeded control values. In the case of *LRP1*, the pattern of changes was identical to that of *RAGE*, except for the 2nd day post-ischemia, where an increase in expression was observed. In other words, the pattern of changes in the expression of both genes post-ischemia from day 7 to 2 years was identical. Changes in *LRP1* and *RAGE* expression resemble a slowly progressive age-related phenomenon.

Two days after ischemia, we found a statistically significant reduction in *RAGE* expression in the frontal cortex, similar to a previous study in the same model in CA3 (Figure 2) [47], which was consistent with the reduction in RAGE in brain and serum after focal cerebral ischemia in rats [48]. Furthermore, within 7–30 days post-ischemia, *RAGE* expression had negative values in the frontal cortex and positive in the CA3 region. Additionally, at the same time, *MAPT* and *SNCA* expression were increased in the CA3 area, with the opposite pattern of changes in the frontal cortex. Thus, in both structures, the pattern of changes was reversed, with a predominance of negative influences in CA3 and positive influences in the frontal cortex.

Significant overexpression of *RAGE* 1–2 years after ischemia suggests its possible role in neuronal death (Figure 2) [49,50,51]. This is indicated by RAGE-positive dying neuronal cells following temporary forebrain ischemia in gerbils [50]. This correlates with previous studies in this model, which demonstrated acute and chronic neuronal changes and progressive neuronal death in the frontal cortex following ischemia [9,17,42]. Furthermore, tau protein, α-synuclein, apoptosis, autophagy, and mitophagy genes have been shown to be associated with progressive ischemic neurodegeneration in the frontal cortex [43,44,45]. Also, ischemia-activated neuroglial cells in the frontal cortex triggered progressive neuroinflammation during a 2-year follow-up [14,15,42]. Additionally, immunohistochemical studies of post-ischemic brains with survival up to 1 year detected the presence of amyloid in and around blood vessels and neurons [9]. In contrast within 1–2 years after ischemia, *RAGE* expression was elevated in the frontal cortex and in CA3 was below control values [47]. Thus, the expression pattern of *RAGE* post-ischemia in the frontal cortex and CA3 region is different, with overexpression in the frontal cortex dominating in the long-term survival groups, which may indicate a negative impact of *RAGE* on long-term survival.

*RAGE* overexpression following cerebral ischemia has been shown to be modulated by hypoxia-inducible factor 1α [52]. It has been noted that excessive expression of neuronal *RAGE* and increased levels of its protein increase the susceptibility of the brain to ischemic damage [53]. Another study showed that RAGE mRNA and protein levels were increased in neuronal cells in the mouse brain after local ischemia [52]. This study also showed that inhibition of *RAGE* signaling resulted in neuroprotection [52]. Additionally, RAGE has been shown to mediate post-ischemic brain injury by inducing neuroinflammation and synaptic dysfunction in an amyloid environment [53,54,55]. In light of the above facts, it is suggested that the neuroinflammatory pathway driven by the RAGE-amyloid interaction may be one of many mechanisms of the development of post-ischemic brain neurodegeneration of the Alzheimer’s disease type [55]. Furthermore, it is known that RAGE induces amyloid production and neurotoxicity in neuronal cells and the transport of amyloid across the blood–brain barrier to the brain tissue [38,56,57,58,59]. Taking into account the fact that significant overexpression of *RAGE* occurs 1–2 years post-ischemia (Figure 2), it can be assumed that it has a significant impact on the development of Alzheimer’s disease-type pathology [60,61]. Overexpression of *RAGE* increases amyloid deposition [58] and apoptosis in the brain, causing cognitive impairment in a mouse model of Alzheimer’s disease [62], suggesting that the same factors likely participate in the development of post-ischemic brain neurodegeneration.

It is now known that ischemia-induced brain neurodegeneration is a type of tauopathy [10,63,64,65,66,67,68,69]. Some data indicate that in neuronal and microglial cells, RAGE binds to tau protein, which facilitates the development of tau protein-related pathologies in cells and behavioral deficits [70]. RAGE also promotes tau protein hyperphosphorylation by activating GSK3 [71,72]. RAGE has also been shown to influence the propagation of transsynaptic tau protein in neurons and to trigger an inflammatory response in microglial cells [70]. Furthermore, it has been shown that overexpression of *RAGE* in neurons underlies the transmission/spreading of tau protein throughout the brain tissue [70]. It has also been shown that amyloid in the brain, the presence of which is guaranteed by RAGE, is a factor triggering the formation of tau protein oligomers [73]. It has been shown that amyloid accumulation in brain tissue can also increase the expression of *RAGE*, which serves as a key receptor in the development of Alzheimer’s disease-like brain neurodegeneration following ischemia [74,75]. Another study showed that RAGE mediates amyloid generation in a mouse model of Alzheimer’s disease by modulating β- and γ-secretase activity [76]. Blocking the AGE/RAGE signaling pathway in the brain has been shown to have a beneficial effect on post-ischemic alterations [77].

Furthermore, previous studies have revealed a crucial role of neuronal and microglial RAGE in ischemia-induced neuronal death and neuroinflammation in irreversible local cerebral ischemia [78,79,80]. On the other hand, RAGE has been shown to induce blood vessel damage, suggesting that RAGE may cause delayed neuronal death as a result of circulatory disruption [80,81]. Furthermore, the above suggestion is supported by a study showing that ischemia and hypoxia trigger endothelial cell pyroptosis via the HIF-1α-RAGE-NLRP3 signaling pathway, resulting in permanent microcirculation injury [82]. It is worth adding that *RAGE* KO mice showed significantly reduced neuronal death post-ischemia, as well as significantly reduced neuroinflammation and blood vessel injury [81]. Another paper described a direct role of neuronal RAGE in promoting ischemic brain pathology in mice [53]. In dominant negative *RAGE* mice, a reduced infarct volume was observed, confirming that *RAGE* signaling is directly linked to post-ischemic brain neurodegeneration [53]. Interestingly, *RAGE* was activated in hypoxic macrophages [83], suggesting that RAGE may also be involved in innate immunity. In this context, RAGE and its ligand HMGB1 have been found to cause brain damage due to ischemia induced by infiltrating macrophages [54]. The role of *RAGE* in promoting macrophage penetration in *RAGE*-deficient bone marrow animals was investigated and revealed that this combination decreases infarct volume following local cerebral ischemia [54]. Inhibition of *RAGE* activity also reduced neuroinflammation, oxidative stress, apoptosis, infarct size, and neurological deficits after local cerebral ischemia [84].

Previous studies have revealed intramembrane proteolysis of LRP1 following cerebral ischemia by γ-secretase, which results in neuronal death [85]. The above data coincide with significant reduction in *LRP1* expression noted in our study 7 days and 0.5 years post-ischemia (Figure 1). The above data do not coincide with the oscillation of *LRP1* expression around control values in the CA3 area after ischemia [47]. LRP1 has been shown to bind amyloid and participate in its removal from brain tissue across the blood–brain barrier [86,87]. The above observations are consistent with a significant increase in *LRP1* expression in our study at 2 days and 1.5 and 2 years in post-ischemic brain injury (Figure 1). In contrast, increased *LRP1* expression was observed in the CA3 region within 1–2 years after ischemia [47]. It is worth noting that a 0.5-year delay in *LRP1* expression was observed in the frontal cortex compared with CA3 within 1–2 years of ischemia [47]. Despite this, the pattern of changes in autophagy, mitophagy, and apoptosis gene expression associated with post-ischemia neuronal death is identical within 1–2 years [43]. The pattern of changes in tau protein and α-synuclein genes, also associated with neuronal death, is also identical but after 1 and 1.5 years of ischemia [45]. Temporal differences in *LRP1* expression in the frontal cortex and CA3 do not appear to significantly influence the eventual development of degenerative changes in these structures after ischemia.

Furthermore, LRP1 is an endocytic receptor that transports ligands from the cell surface to the endosomal compartment, where these ligands are sorted into the lysosomal compartment and degraded. The above-mentioned LRP1 mechanism has been shown to regulate the internalization, degradation and spread of tau protein in brain tissue lysates from Alzheimer’s disease patients [38], suggesting that this phenomenon is likely to also occur in post-ischemic neurodegenerative processes. This observation identifies LRP1 as an endocytic receptor that binds, transports and contributes to the processing of monomeric forms of tau protein, which consequently leads to its degradation and ultimately prevents its seeding [38]. The balance of these processes is likely crucial for the propagation of neuropathology in our ischemic model of Alzheimer’s disease [38]. LRP1 has also been shown to be a master regulator that interacts with heparan sulfate proteoglycans, thereby controlling tau protein entry into neurons [39].

LRP1 has been shown to attenuate oxidative stress, neuroinflammation, and apoptosis and to reduce short- and long-term neurological deficits and mortality following brain ischemia in mice through inhibiting the TXNIP/NLRP3 signaling mechanism [88]. It was shown that the activity of the LRP1/TXNIP/NLRP3 mechanism was significantly increased within 2–5 days after ischemic brain injury [88]. This process coincided with a significant increase in *LRP1* expression in our study 2 days post-ischemia. Moreover, LRP1 has been shown to exert its neuroprotective effects through interaction with apolipoprotein E [88], whose gene we found overexpressed in the frontal cortex of our model before day 7 and after 1–2 years of survival following cerebral ischemia [44].

LRP1 has been shown to positively influence neuropathogenesis after local brain ischemia through its anti-apoptotic activity [89,90]. A protective effect of LRP1 after local cerebral ischemia has also been demonstrated, via mitochondrial interaction between astrocytes and neurons. Using cell culture and an animal model of regional cerebral ischemia, it was demonstrated that astrocytic LRP1 regulated the transfer of healthy mitochondria from astrocytes to neurons and protected neurons from ischemia–reperfusion injury [91]. Inhibition of astrocytic LRP1 activity reduced mitochondrial transfer to injured neurons and impaired post-ischemic recovery [91].

Data from this and previous studies indicate unusual changes in gene expression that begin around the first year after ischemia, but we currently do not know why this occurs or what this means [43,44,45,47]. From previous studies using this model, we know with certainty that neuronal changes and death develop slowly in various brain structures over a two-year period [9,17,19,42]. This leads to generalized brain atrophy and the development of Alzheimer’s disease-type dementia [19]. The effects of increased *RAGE* expression in the frontal cortex 1–2 years after ischemia are likely mitigated by the influence of the natural increase in *LRP1* expression. This indicates an opposing effect of both genes on long-term survival after ischemia, with the negative effect of *RAGE* predominating. It should be noted that this naturally occurring phenomenon cannot currently be fully explained and further research into its mechanisms and persistence is necessary. This requires elucidation of all mechanisms at the genomic and proteomic level and their interactions and duration related to the early and late stages after ischemia. It appears that the balance of these mechanisms may be crucial for the spread of neurodegeneration in the brain after ischemia. The data indicate that these two genes influence damage to the frontal cortex, representing an additional, previously undescribed pathological phenomenon in the form of neuronal damage and death in this region, contributing to impairment of whole-brain function. These data correlate with previously published acute and chronic neuropathological changes in the cerebral cortex post-ischemia with 2 years survival [9]. Furthermore, they correlate with previously demonstrated memory impairment and the development of dementia in these rats following ischemia [19].

It is likely that this time (1–2 years) after ischemia determines whether the changes will progress toward sustained survival or irreversible changes. Furthermore, our study shows a clear trend toward the predominance of *RAGE* expression, which would constitute a negative effect. Currently, we lack data or guidance on how to interpret these observations and what they actually mean in relation to post-ischemic survival; therefore, further, highly precise studies using advanced methods are necessary. Finally, it should be emphasized that due to the fact that the studies were conducted on female rats, the obtained results cannot be directly translated to representatives of both sexes, which is a significant limitation of these studies.

A limitation of our study was the small number of rats in each group. The small number of rats at each time point resulted from restrictions imposed by the Bioethics Committee, which limited the number of animals and the availability of research material. Furthermore, this has limited the scope of the research, particularly on protein levels in the frontal cortex and blood, to assess how this relates to changes in gene expression. Additionally, this limited the possibility of conducting additional studies on males to compare the effects of ischemia on sex. Also, the conclusions should be supported by studies on local and total cerebral ischemia in various animal species, regardless of gender. The cells in which the expression of the studied genes predominates also require clarification, so it is necessary to conduct studies on single cells after ischemia. Furthermore, it is necessary to determine the impact of gene expression changes on cellular phenomena such as reversible and irreversible damage, neurodegeneration in association with aging, the course and severity of neuroinflammation, mitochondrial health, and the development of oxidative stress, as well as toxic effects of amyloid and tau protein aggregates. This would enable a more accurate interpretation of the data and the identification of the turning point mechanisms in the transition from acute to chronic changes after ischemia.

## 4. Materials and Methods

### 4.1. Animals and Brain Ischemia

Female Wistar rats (n = 70, 130–150 g) were subjected to 10 min global cerebral ischemia with survival at 2, 7, and 30 days and 0.5, 1, 1.5, and 2 years [92]. Just before performing brain ischemia, anesthesia with 2% isoflurane with oxygen was discontinued. Transient brain ischemia was achieved by cardiac arrest [92]. A hook made of an L-shaped steel needle was introduced into the chest by the right parasternal line and the third intercostal space and placed under the vascular bundle of the heart [92]. Next, the hook was gently moved towards the spine. Subsequently, the hook was gently tilted 20° towards the tail, and this meant that the hook in this position was under the heart vessels. The hook was next pulled to the sternum, which led to closure of the heart vessel bundle through the sternum. In order to prevent chest movements and ensure closure of the heart vessels, external pressure was applied to the sternum with the index and middle fingers, which resulted in complete hemostasis and cardiac arrest [92]. After 10 min, the hook was removed from the chest and resuscitation started. Resuscitation started with artificial ventilation and external heart massage until spontaneous heart activity returned and breathing. During this time, air was administered using a respirator [Ugo Basile, Gemonio, Italy] through a polyethylene tube inserted into the trachea. The heart massage frequency was 150–240/min. Females rats were chosen because they survived without problems for up to 2 years after ischemia, and as we know Alzheimer’s disease is more common in women. Animals that were successfully resuscitated were housed in a specially designated room with a separate entrance, in pairs, in cages, isolated from other animals not participating in the experiment, and remained under the care of the same person throughout the observation period. Under these conditions, we observed no mortality during the two-year survival period, provided there was no contact with other rats or bystanders and the rooms were air-conditioned. Over time, signs of aging were clearly visible in the animals, such as slower movement, dozing, poorer fur quality (duller, sparser), changes in body weight, slower responsiveness to stimuli, and behavioral changes, with the severity of changes being more pronounced in the post-ischemic group than in the sham group. Both the post-ischemic and control groups consisted of 70 animals each. As mentioned, no animals died, and none were excluded from the study. Survival in both groups was 2 days (n = 10), 7 days (n = 10), 30 days (n = 10), 0.5 years (n = 10), 1 year (n = 10), 1.5 years (n = 10) and 2 years (n = 10). Animals in the control group were subjected to identical procedures except for cardiac arrest. In both groups, there were 10 rats for each survival time. The animals used in the study were kept in cages of two in a room with a temperature of 21 ± 1 °C, air humidity of approximately 50% and a 12 h light–dark cycle. All experiments were completed during the day. During the study, the animals had free access to water and food. Rats in the studies were treated in accordance with the NIH Guide for the Care and Use of Laboratory Animals (1985), European Communities Council Directive 142/86/609/EEC, and with the approval of the local Ethics Committee (No. 53/2014 of 16 January 2015). All efforts were made to minimize animal suffering and to reduce the number of rats used.

### 4.2. Sample Preparations

After the studies were completed, the brains were perfused with cold 0.9% NaCl through the heart. Then, after removing the brains from the skull, samples of the frontal cortex with a volume of approximately 1 mm^3^ were taken and placed in RNALater solution (Life Technologies, Carlsbad, CA, USA) [93]. The frontal cortex samples were homogenized in 1 mL of TRI-Reagent buffer for RNA isolation (Ambion, Austin, TX, USA). The suspension was then incubated for 5 min at ambient temperature, after which 200 μL of chloroform (Sigma-Aldrich, St. Louis, MO, USA) was added and the samples were shaken for 15 s. In the next step, the sample was incubated for 15 min at ambient temperature, then centrifuged for 15 min at 14,000 rpm. Subsequently, 500 μL of 2-propanol (Sigma-Aldrich, St. Louis, MO, USA) was added to the aqueous fraction. The parts were then mixed and incubated for 20 min at ambient temperature. Samples were centrifuged again for 20 min at 14,000 rpm at 4 °C. The RNA part was then placed in 80% ethanol and stored at −20 °C.

### 4.3. Quantitative PCR

*LRP1* and *RAGE* expression was assessed by reverse transcription-quantitative polymerase chain reaction (RT-qPCR) [90]. RNA qualitative and quantitative parameters were assessed using a NanoDrop 2000 spectrophotometer (Thermo Scientific, Waltham, MA, USA) [93]. For the study, 1 μg of RNA was used, which was reverse transcribed into cDNA using a high-capacity cDNA reverse transcription kit (Applied Biosystems, Foster City, CA, USA). cDNA synthesis was performed using Veriti Dx (Applied Biosystems, Foster City, CA, USA) in the following steps: step I 25 °C, 10 min; step II 37 °C, 120 min; step III 8 °C, 5 min; step IV 4 °C. The cDNA was then amplified by real-time gene expression analysis (qPCR) on a 7900HT Real-Time Fast system (Applied Biosystems, Foster City, CA, USA) [93]. Commercial TaqMan FAM-MGB probes from ThermoFisher Scientific Inc. (Waltham, MA, USA) were used to study gene expression. LRP1 gene: Catalog number: 4331182; ID: Rn01503901_m1; Chromosome location: Chr.7: 70846313–70927028 on Build Rnor_6.0. RAGE gene: Catalog number: 4331182; ID: Rn01430278_m1; Chromosome location: Chr.6: 135228755–135259746 on Build Rnor_6.0. The studied genes were normalized to the control gene Rpl13a. Rpl13a gene: Catalog number: 4331182; ID: Rn00821946_g1; Chromosome location: Chr.1: 101120711–101123401 on Build Rnor_6.0.

The studied genes in the ischemic and sham groups were related to the control gene Rpl13a. The relative quantity (RQ) of the studied genes was estimated using the ΔCT method, and the values are presented as RQ = 2^−ΔΔCT^ [93]. The final values are presented using logarithmic conversion of RQ values (LogRQ) [93]. LogRQ = 0 means that the tested genes did not change with respect to the control. LogRQ < 0 indicates decreased gene expression and LogRQ > 0 indicates increased gene expression after ischemia compared to the control group.

### 4.4. Statistical Evaluation

Statistica v. 12 was used for statistical evaluation of the data, using the nonparametric Kruskal–Wallis test with the Z test for multiple analysis of differences between groups. Data are presented as mean ± SD. *p* ≤ 0.05 was used to determine statistical variability.

## 5. Conclusions

Based on the obtained results, it can be concluded that changes in *LRP1* and *RAGE* in the time interval from 2 days to 0.5 years of life post-ischemia probably do not have a significant impact on the course of pathological processes. However, data showing superiority of *RAGE* expression over *LRP1* expression 1–2 years after ischemia may suggest a predominance of negative effects associated with *RAGE* expression. The precise role of *LRP1* and *RAGE* in the development and progression of post-ischemic neurodegeneration, as well as the temporal and mechanistic conditions under which they exert protective or negative effects following ischemic brain injury, remains unclear. Despite the expanding knowledge base, further studies are necessary to elucidate the precise processes underlying the time-dependent and region-specific responses of *LRP1* and *RAGE*.

## Figures and Tables

**Table 1:** Summary of *LRP1* and *RAGE* expression data at different times after brain ischemia.

Gene	Time	N	Mean	Median	Minimum	Maximum	SD
*LRP1*	2 days	10	0.686	0.641	0.413	1.195	0.248
*LRP1*	7 days	10	−0.354	−0.420	−0.638	−0.042	0.208
*LRP1*	30 days	10	−0.231	−0.092	−1.010	−0.061	0.293
*LRP1*	6 months	10	−0.546	−0.551	−0.860	−0.353	0.156
*LRP1*	12 months	10	0.187	0.100	0.066	0.460	0.153
*LRP1*	18 months	10	0.940	0.943	0.127	1.817	0.720
*LRP1*	24 months	10	0.293	0.208	0.070	0.711	0.238
*RAGE*	2 days	10	−0.511	−0.460	−0.943	−0.181	0.268
*RAGE*	7 days	10	−0.407	−0.416	−0.824	−0.080	0.241
*RAGE*	30 days	10	−0.135	−0.093	−0.314	−0.041	0.098
*RAGE*	6 months	10	−0.586	−0.561	−0.735	−0.441	0.107
*RAGE*	12 months	10	1.342	1.749	0.268	2.178	0.773
*RAGE*	18 months	10	0.212	0.210	0.062	0.377	0.099
*RAGE*	24 months	10	0.823	0.762	0.316	1.401	0.347

N—number of samples, SD—standard deviation.

## Data Availability

The data of this study can be made available on request from the corresponding author.

## Abbreviations

The following abbreviations are used in this manuscript:

LRP1	low-density lipoprotein receptor-related protein 1
N	number of samples
RAGE	receptor for advanced glycation end products
RQ	relative quantity
RT-PCR	reverse transcription-quantitative polymerase chain reaction
SD	standard deviation
